# Clearing the trichiasis backlog: experiences in Amhara, Ethiopia

**Published:** 2015

**Authors:** Esmael Habtamu, Matthew Burton

**Affiliations:** Trachoma Research Program Officer: The Carter Center, Atlanta, USA.; Reader: London School of Hygiene and Tropical Medicine, London, UK.

**Figure:**
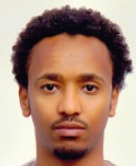
Esmael Habtamu

**Figure:**
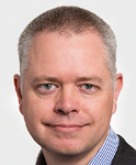
Matthew Burton

Globally, there were estimated to be around 7.3 million people in 2009 with trachomatous trichiasis (TT).^1^ The World Health Organization (WHO) recommends surgery to reduce the risk of sight loss.^2^ In the last decade, only about 50% of the annual global surgical targets have been achieved.^3^ At the current rate, it has been estimated that the trichiasis backlog would not be adequately addressed until 2032, twelve years after the Global Elimination of Trachoma (GET) 2020 target date to eliminate blinding trachoma.^4^

Deploying community-based screening and counselling using dedicated mobile surgical teams in high-burden districts, integrating with other eye care services and leveraging political support at all administrative levels are among potential solutions to improve trichiasis surgical uptake.

## Challenges of surgical uptake

In the Amhara Region of Ethiopia it was recognised that many trichiasis patients were not accepting offers of surgery through local, freely provided outreach services. This was explored in focus group discussions, which identified a variety of concerns. First, most trichiasis patients mistakenly believed that the surgical wound needs up to 2 months to heal, during which time they should avoid sunlight exposure, involvement in productive activities and getting near to fire or smoke, or else TT would recur. Patients also believed that the operation was very painful and were unaware that it is conducted under local anaesthesia. They were very concerned about the quality of surgery. Consequently, patients either tended to decline surgery (even after presenting to local surgical sites) or chose surgical services provided by external surgical teams over the locally available surgical services. Despite these concerns, and apparently incomplete patient knowledge, surgical programmes tended to focus their ‘patient mobilisation’ efforts (efforts to improve the uptake of services) on simply creating awareness of surgical services; they did not engage sufficiently with communities to address their concerns.

**Figure 1.**
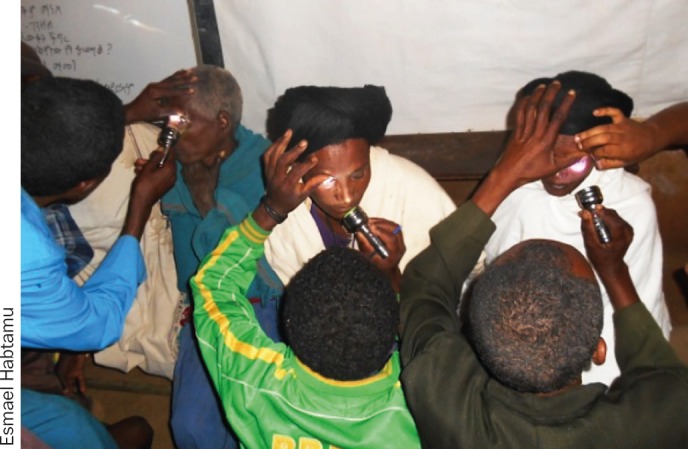
Eye ambassadors being trained and screening patients

## A discussion-based approach

It is essential to address the concerns of trichiasis patients and their relatives through discussion and counselling. In Amhara, community-based screeners (eye ambassadors) have been trained to identify, counsel and refer patients. They visit all households in their target villages and examine everyone aged 15 years and older using a torch. If people are identified as having TT, eye ambassadors talk with them and their relatives to address their concerns and correct misunderstandings. The patient is then referred to the nearest health facility for examination and further counselling (by trichiasis surgeons).

There is some evidence that this discussion-based approach is working. In a cluster of six large villages where 43 eye ambassadors identified and counselled patients, 240 operations were performed within the following two weeks. In an adjacent cluster of villages, five outreach visits were organised at the same time using the usual mobilisation methods (sending messages as public announcements in the market and through health extension workers); this resulted in just one patient undergoing an operation. In a third cluster, an outreach visit organised using just public announcements in the market led to no patients coming for surgery. However, when the eye ambassador approach was used in the same cluster a short while later, 114 patients underwent surgery in less than a week.

## Service delivery models

Different service delivery models can be adapted based on local needs and the availability of resources. In many trachoma endemic countries, there are insufficient trichiasis surgeons to clear the current trichiasis backlog using traditional static and outreach models. To achieve GET 2020, particularly in high-burden areas, more productive approaches are needed. One approach is to deploy a dedicated mobile team of trichiasis surgeons and ancillary staff to travel to districts of high trichiasis burden. With good coordination and communication with communities in need, hundreds of operations can be conducted by a single team within a few weeks. In Amhara, our mobile team performed more than 1,378 trichiasis operations within three months. In addition, the quality of surgery is likely to be higher due to the high volume and the increased opportunities for supportive supervision. Community-based trichiasis screeners and counsellors can help to provide pre- and post-operative support and services.

## Political will

Gaining political support at all administrative levels is needed. At the GET 2020 meeting held in Addis Ababa in 2014, the Ethiopian Health Minister made it a personal mission to clear Ethiopia's trichiasis backlog within 18 months. He announced a fast-track initiative and provided additional funding for surgeons.^5^ This has provided political impetus and set a good example for the global trachoma community. At the district level, the identification and counselling of trichiasis patients and the establishment of mobile surgical teams require similar support from local political leaders.

One of the biggest challenges in clearing the trichiasis backlog is convincing trichiasis patients to accept surgery. Investing in developing surgical teams without ensuring patient uptake of surgery would be wasteful. Training and deploying community-based screeners appears to be a very effective method.
